# A perspective on SIDS pathogenesis. The hypotheses: plausibility and evidence

**DOI:** 10.1186/1741-7015-9-64

**Published:** 2011-05-27

**Authors:** Paul N Goldwater

**Affiliations:** 1 Microbiology & Infectious Diseases, SA Pathology at the Women's & Children's Hospital; 2 Discipline of Paediatrics, University of Adelaide, 72 King William Road, North Adelaide, SA, Australia

## Abstract

Several theories of the underlying mechanisms of Sudden Infant Death Syndrome (SIDS) have been proposed. These theories have born relatively narrow beach-head research programs attracting generous research funding sustained for many years at expense to the public purse. This perspective endeavors to critically examine the evidence and bases of these theories and determine their plausibility; and questions whether or not a safe and reasoned hypothesis lies at their foundation. The Opinion sets specific criteria by asking the following questions: 1. Does the hypothesis take into account the key pathological findings in SIDS? 2. Is the hypothesis congruent with the key epidemiological risk factors? 3. Does it link 1 and 2? Falling short of any one of these answers, by inference, would imply insufficient grounds for a sustainable hypothesis. Some of the hypotheses overlap, for instance, notional respiratory failure may encompass apnea, prone sleep position, and asphyxia which may be seen to be linked to co-sleeping. For the purposes of this paper, each element will be assessed on the above criteria.

## Background and leading hypotheses

Before committing to this task it is apposite to consider the background and context in which the various hypotheses arose.

Beckwith's 1970 definition of SIDS [1] created the presumption that SIDS babies were normal. Questions arose after Kinney *et al*. [2] developed their hypothesis based on histopathological abnormalities of the brain, a prenatal origin of these and sudden death occurring during a vulnerable period in infancy. Bergman (1970) [3] had argued against a "single characteristic that ordains an infant for death," but for an interaction of risk factors with variable probabilities. This approach was supported by Wedgewood (1972) [4] whose hypothesis consisted of host vulnerability, age-specific risks, and precipitating factors. Others have supported a multifactorial approach (Raring 1975) [5], (Rognum and Saugstag 1993) [6].

The triple risk hypothesis of Filiano and Kinney (1994) [7] has been popular with its central focus on brainstem prenatal injury in a subset of SIDS, denoting a not universal finding. The National Institute of Child Health and Development SIDS Strategic Plan 2001 [8], stated unequivocally that 'Knowledge acquired during the past decade supports the general hypothesis that infants who die from SIDS have abnormalities at birth that render them vulnerable to potentially life-threatening challenges during infancy.' In essence, this states that SIDS is a developmental disorder originating during fetal development. Interest in the brainstem began with Naeye's (1976) [9] findings of astrogliosis in 50% of SIDS and controls. Hypoxia was considered to be the underlying cause. Kinney *et al. *(1983) [10] found gliosis in only about one-fifth of SIDS cases. This shortfall stimulated studies on brainstem neurotransmitters [11,12]. Ambler *et al. *(1981) [13] could not distinguish SIDS from other deaths on the basis of neuropathological findings and concluded that known causes of sudden death were unsuitable as a control group. Considerable debate over the time of onset of the brainstem changes continues to this day (this will be discussed in more detail).

Reviewing the triple risk hypothesis, Guntheroth and Spiers (2002) [14] would not support a categorical statement on the origin of SIDS being prenatal and concluded that the brainstem abnormalities have not been shown to cause SIDS but are more likely to be a non-specific effect of hypoxia. (This argument will be revisited).

A quite separate approach to SIDS pathogenesis, while containing the fundamental vulnerability of the SIDS infant, age-specific risks, and precipitating factors is the common bacterial toxin hypothesis developed by Morris (1999) [15]. In this we find evidence of the existence of bacterial toxins of common bacteria (for example, *Staphylococcus aureus, Escherichia coli*) in SIDS cases compared with healthy babies. The hypothesis suitably accounts for epidemiological features of SIDS (including a mathematical model) and contains plausible explanations of the pathological findings (Goldwater) [16]. This area of SIDS research having compiled considerable supportive evidence has had difficulties in achieving broad acceptance for reasons that are not immediately apparent but seem to be sustained by opinion outside scientific reason. The work is well supported by the findings of groups led by Blackwell [17-19], Morris [20,21], Smith [22], Bettelheim [23-26], Murrell [27] and Goldwater [16,26,28-31]. Goldwater's group extended the hypothesis to encompass inherent or acquired deficiency in pathogen recognition [32], which is predicated on the findings of significant sterile site bacterial infection [33,28], which is proposed to represent a footprint of commonly occurring bacteremic events of infancy and childhood [34] from which most individuals achieve uncomplicated recovery. There is no reason to exclude from the hypothesis a role of viral triggers acting in concert with bacterial products. Direct and indirect (that is, cytokine) evidence of viral infection in SIDS is well known and this with hypoxaemia has been a focus of Rognum's group [6]. Other hypotheses include cardiogenic causes proposed by Poets [35,36] and Matthews [37], shock including anaphylaxis [38,39], and thermal stress [40-43].

## Other hypotheses

Among the less convincing hypotheses for which evidence is poor are the following: 1) CO2 rebreathing [44,45]: Guntheroth and Spiers [43] argued that some of the risk factors for rebreathing could be explained by the effects of thermal stress, several factors for thermal stress could not reasonably be explained by the rebreathing hypothesis; 2) toxic gases emitted from fungally colonized mattresses [46], and abuse [47] and unintentional asphyxia [48], and 3) respiratory tract obstruction. Upper respiratory obstruction has long been promulgated by Krous [49], Tonkin [50], and others based on purported anatomical changes said to occur when a baby is placed prone. While some supportive evidence exists [51], such a mechanism is undermined by the lack of evidence that asphyxiation is a plausible mode of death in SIDS.

## Discussion

### **The pathological findings in SIDS**

In order to fully examine hypotheses of SIDS causation, it is essential to acknowledge the pathological findings in SIDS. These hypotheses have too often been limited to one organ site or have been overlooked. This is surprising and extremely unsatisfactory. Many pediatric pathologists expert in Sudden Unexpected Death in Infancy (SUDI) maintain a stance that deaths from asphyxia are indistinguishable on gross pathology from SIDS without providing supportive evidence. To the contrary, there is, however, evidence that pathological findings in SIDS are salient and consistent [16,52]. They consist of 1) intrathoracic petechial haemorrhages; 2) thymomegaly, 3) encephalomegaly, 4) evidence of microcardia, 5) liquid unclotted blood in the chambers of the heart, 6) kidney growth-restriction is also well described [53,54] and 7) an empty bladder and rectum [55,56]. Given the fact that most SIDS cases share this common pathological picture, it would, in all probability, be reasonable to suppose these pathological features are the result of a single cause or a related underlying pathophysiology. While seemingly against mainstream consensus, it would seem improbable that such a regular spectrum of pathologies would be the result of a myriad or even a few possible causes. In the absence of these salient findings, in cases where the history and death scene were not congruent with SIDS, experienced pathologists would consider alternative diagnoses. Other more subtle pathologies, which are less consistent in terms of the proportion of SIDS babies affected, include the brainstem changes alluded to above. In 2004 SIDS categories were developed based on clinical, death scene, and autopsy information [57]. For the purposes of this paper, while acknowledging this formalization of the redefinition, there is little to be gained by a focus on this. Regardless of the definition (of which we all are aware is one of exclusion) it is not helpful in discussing the essence of my thesis which is this; Because there is such a consistent pathological picture (three-site intrathoracic petechiae, liquid blood, high brain weight, and so on) found in approximately 90% of cases of those deemed SIDS, the statistical chance of the constellation of pathological features occurring in approximately 90% of cases being caused by separate pathogenetic mechanisms is infinitesimal. This being so would, therefore, justifiably be explained by a single pathogenetic process. In cases where there is a possibility of the existence of, for example, prolonged QT syndrome as being a cause of SIDS (a situation not thought likely to be involved in most cases of SIDS (on the basis of the low rate of genetic predisposition), such cases might fit into the approximately 10% of cases not exhibiting the typical constellation of pathological findings. Unfortunately, there is no study of which I am aware that has reported the pathological findings in such cases. Moreover, there is a general lack of attempt to characterize pathology with the various proposed causes of SIDS.

### Petechial haemorrhages

Petechial haemorrhages in SIDS are small spot haemorrhages of unknown aetiology found on the surfaces and within the tissues of intrathoracic organs. Their presence is regarded by some pathologists as a pre-requisite for making the diagnosis of SIDS [58], while Hilton is quoted [59] that petechiae "are never present on the conjunctiva, eyelids or on or in other soft tissues of the head or neck in SIDS." Byard and Krous [60] support the contention that finding petechiae on the face, neck, upper chest, or conjunctivae warrants suspicion and extremely careful investigation.

Petechial haemorrhages in/on intra-thoracic organs (thymus, lungs, visceral pleura and epicardium) are an almost universal finding in SIDS at autopsy; however, they may also be found, but less frequently, in other infant deaths. In non-SIDS, the numbers of petechiae are generally fewer and the distribution less frequently involves all three intrathoracic organs. Table 1 provides comparative data from previously published studies. Until the analysis by Goldwater [61] and Kleeman *et al. *[62] delineation of the differences between SIDS petechiae and those found in asphyxial and other deaths had not been satisfactorily addressed in relation to unexplained infant deaths.

**Table 1:** Incidence of intrathoracic petechial haemorrhages in SIDS and comparison cases

No. SIDS cases	Frequency of petechiae	Non-SIDS comparisons	Reference
31	80%	absent or sparse in infant suffocation, CO asphyxia, drowning	Werne & Garrow [152]
12	100%	none	Handforth [153]
97	95%	rare in infanticide, accidents	Jacobsen and Voight [154]
80	79%	6 of 43 (14%)	Geertinger [155]
91	94%	10 of 31 (32%) in no case were they numerous	Cooke&Welsh [156]
162	68%	12 of 42 (29%)	Marshall [157]
109	87%	16 of 38 (42%)	Beckwith [158]
100	85%	none	Krous [68]
622	82%	39 of 65 (60%)	Valdes-Dapena *et al*. [159]
63	87%	13 of 33 (39%)^#^	Risse & Weiler [67]
174	62.7* to 89.55%^#^	18 of 67 (26.8%)^+ ^- 31 of 67 (47.7%)**	Goldwater [61]
311	70%^#^	42 of 80 (52.5%)^#^	Fracasso *et al*. [79]
250	91%	29 of 69 (42%)	Kleeman *et al. *[62]

* ^+ ^% with petechiae present in all three organs; ^# ^** % with petechiae present in/on thymus

### Pathological correlates: Real or not?

#### Petechial haemorrhages, brainstem changes and SIDS

A number of authors have attempted to link brainstem changes with intrathoracic petechial haemorrhages. For example, Waters' group [63] investigated association of certain aspects of brainstem pathology (for example, apoptosis detection using Terminal Transferase and Biotin-16-dUTP (TUNEL staining) with modifiable risk factors of SIDS (a history of cigarette smoke exposure and the sleep-related parameters of bed sharing and prone sleeping) and included petechiae, and blood in and around the nose [63]. Waters' group showed positive associations with abnormal brainstem pathology and modifiable SIDS risk factors were only observed for TUNEL staining. These included the presence of petechiae, and blood in or around the nasal area. Not surprisingly the authors found intrathoracic petechiae significantly more frequent in SIDS than controls and noted that 'the presence of petechiae is a common pathological finding amongst SIDS infants but is not conclusive to all SIDS cases, and is observed in infants dying from other causes' [64].

While the cause of petechiae in SIDS is still unknown, there is some evidence that their distribution and frequency is affected by the age, ethnicity, parity, exposure to cigarette smoke and the sleep position of the infant [64,65] the latter leading to the suggestion that the petechiae arise from an obstruction of the airways [65]. Krous *et al*. [65] claim that 'since upper airway deaths and SIDS cases share a similar distribution of petechiae, it seems reasonable to postulate that airway obstruction might occur in SIDS.' Inconsistencies can be seen in this approach; on the one hand, the number, density and distribution of intrathoracic petechiae between the two conditions have been shown to be reasonably distinctive [64], whereas on the other hand, respiratory obstruction is frequently accompanied by extrathoracic petechiae. Intrathoracic petechial hemorrhages were encountered by Beckwith in 87% of SIDS cases but in non-SIDS cases 'were mostly absent or less developed in quantitative terms' [66]. Others have similarly commented [67,68].

A comparison of the distribution of petechiae in SIDS and various other causes of death showed, with few exceptions, limitation to within the chest cavity in SIDS but extension to below the diaphragm in infants in whom the terminal course was complicated by hypoxaemia, hypercarbia, metabolic acidosis, coagulopathy, or infection [68]. Interestingly, and perhaps because the comparison (SUDI) deaths in the study by Goldwater [61] were selected for having died suddenly, petechiae were rarely encountered in the conjunctiva, peritoneum or on the surface of abdominal organs. In SUDI occurring as a result of asphyxia/strangulation, skin petechiae on the head, neck and upper torso were observed relatively commonly [68]. Krous *et al. *claim their data support the association with an obstructed airway because of the positive association between the presence of petechiae and increased TUNEL staining in the rostral dorsal motor nucleus of vagus (**DMNV**), of SIDS infants. This proposition could be seen as illogical and its justification on physiological grounds cannot be sustained. Care in interpretation of positive TUNEL staining is required as it should not be considered as a specific marker of apoptosis but can also indicate necrotic cell death [69]. Krous *et al. *found no association was found between presence or density of intrathoracic petechiae and prone (face down) position or age [65]. Despite evidence to the contrary, the authors continued to pursue a 'respiratory' pathogenetic explanation.

While no widely accepted animal model for SIDS has been described, intrathoracic petechiae have been observed at necropsy in rats killed by tracheal occlusion [70] and demonstrated that intrathoracic petechial hemorrhage could be associated with asphyxiation, (pertaining to a rat model). Others have shown similar or even contradictory findings; for example, in newly mature rats that were free of infection, hypoxic asphyxia produced an insignificant number of petechiae, whereas in all littermates infected with an enzootic virus (Sendai) large numbers of petechiae developed with hypoxic asphyxia. Rats similarly infected, but killed with an overdose of pentobarbital sodium, had no petechiae. Most importantly, infected rats with unremitting airway obstruction were free of petechiae. Thus, the experimental conditions necessary for the presence of intrathoracic petechiae were profound hypoxia and infection, with persistent circulation and respiratory effort; persistent airway obstruction *per se *appears not to produce petechiae, with or without infection [71].

Becroft reported the findings on 474 autopsied SIDS cases and found macroscopic petechial hemorrhages in the visceral pleura, capsule of thymus, and epicardium in 458 (96.6%) [64]. Multivariable analysis of this study showed significant associations among increased frequencies of thymic petechiae and parity, age at death, Maori ethnicity, pacifier (dummy) use, and head covering at death. Also significant were associations between increased frequencies of epicardial petechiae and head covering at death and estimated time of death between 00:00 and 05:59 h; and between increased frequencies of pleural petechiae and maternal smoking and parity. There was a decreased frequency of pleural petechiae in infants placed prone for their final sleep with age acting as a possible confounder for the prone sleep position correlation. The distribution and frequency of petechiae seemed to be affected by SIDS environmental (known) risk factors, but these factors occurred inconsistently for the three organ sites. Regrettably, the findings of Guntheroth *et al*. [71] were ignored as there was a failure to examine associations with viral infection without which knowledge their claim that their findings implied differences in the pathogenesis at each intrathoracic organ site cannot be taken seriously.

The findings of Goldwater [61] showed the concurrence of petechiae in all three intrathoracic sites was highly predictive of a SIDS diagnosis and indicated that a common causal mechanism almost certainly underlies the specific site petechiae. The occurrence of triple-site petechiae in non-SIDS deaths was shown to be relatively rare. Extrathoracic petechiae occurred almost exclusively in non-SIDS cases. Significant differences were observed between the SIDS and non-SIDS groups: the presence of thymic petechiae in SIDS (89.5%) compared with non-SIDS (47.7%) was highly significant (*P *< 0.000001; OR 9.38 (CL 4.5 to 19.9), and their absence was more frequent in non-SIDS (52.3%) compared with SIDS cases (10.4%) (*P *< 0.000001). Pleural petechiae were found in 80% SIDS and 47.5% non-SIDS (*P *= 0.000002; OR 4.6 (CL 2.3 to 9.1)). Epicardial/cardiac petechiae were found in 79.9% SIDS and 43.6% non-SIDS (*P *< 0.000001; OR 5.3 (CL 2.6 to 10.8)). Petechiae in all three sites (thymus, pleura, heart) were found in 62.7% of SIDS and 26.8% of non-SIDS cases (*P *< 0.000001; OR 4.6 (CL 2.3 to 9.0)). Of note is the high predictive value of finding petechiae in all three intrathoracic sites (positive predictive value 84.9%) and the predictive value of their absence from all three sites (93.1%) in supporting a diagnosis of SIDS or ruling it out, respectively. The findings, showing such a clear difference between the two groups of SUDI almost certainly indicate different underlying pathogenetic mechanisms.

In a study of 473 SIDS cases by Krous *et al. *[72], face position when found was specifically described for 332 (70%). Of 122 cases found face down, 112 (92%) had intrathoracic petechiae, compared to 85% (179) of 210 infants found with the face up or to the side (*P *= 0.06). These data clearly show that hypothetical upper airway obstruction attributable to face-down position is not causally related to development of intrathoracic petechiae. Despite these findings and those of Poets *et al. *[35,36] Kraus *et al. *continue to support an "internal" upper respiratory causation of intrathoracic petechiae.

In conclusion, while anatomical brainstem abnormalities may exist in SIDS brains, the link between these and respiratory function remains tenuous and unproven and the criteria (1. Does the hypothesis take into account the key pathological findings in SIDS? 2. Is the hypothesis congruent with the key epidemiological risk factors? 3. Does the hypothesis link 1 and 2?) remain unsatisfied. Cessation of respiration as a cause of death is often associated with extra-thoracic petechiae which are rarely seen in SIDS. Such a fact tends to undermine the respiratory hypothesis. While many authors claim intrathoracic petechiae are caused by increased intrathoracic pressure associated with respiratory failure, logically we would expect to see accompanying extrathoracic petechiae also. This we do not observe. An alternative mechanism must, therefore, be sought. The answer may lie in the relationship between brainstem anomalies, and a cardiogenic death associated with gasping. These combinations are examined below.

Ambler *et al*. [13] describe the neuropathological findings in 58 infants and children dying suddenly and unexpectedly. Utilizing historical, clinical, laboratory and pathological findings, two subgroups were distinguished: in one, a cause of death was established (CODE); members of the other (more than 50% of the total sample) were SIDS. The importance of historical as well as pathological data in excluding SIDS was stressed. In each subgroup, both focal lesions and diffuse glial reactive hypertrophy were identified in 64% of all children below nine months of age. These changes were not related to age group or maturation and, except for a history of perinatal asphyxia, lesions were not predictably correlated with clinical data. The authors concluded that the brains of children dying of established cause (CODE) are not a suitable control group with which to compare those of SIDS. This begs the question: what is a suitable control group?

#### Thymomegaly, encephalomegaly, microcardia, light kidneys

Considerable debate simmered for many years of theories such as *status thymico-Lymphaticus *[36]. The essence of the theory was lost after revelation of the unconscionable practice of irradiation of children's thymuses. Underlying disease processes that are associated with the organ weight differences between the SIDS and non-SIDS cases include infection, inborn metabolic disorders, genetic mutations, and immune disorders [73]. Some investigators speculate that decreased organ growth may contribute to mortality, while others suggest that the differences in organ weight are caused by inadequate reference weight data. An approach that considers organ weights relative to body weight could obviate the need for revised reference data. Allometric regression analysis allows comparisons without the need for reference data, and has been employed in analysis of a wide array of biological data, specifically the relative growth of brain and body weights among the Order Primates [74], and organ/body weight ratios among SIDS cases [75,76].

When organ weights are measured as a function of body weight (not age) some studies show significant differences between cases of SIDS [77,78] and non-SIDS while others contradict these [79]. Reasons for the discrepancies between the studies relate to features of the control groups (presence of infection, gestational age, changes in infant nutritional status with socioeconomic development, and so on). Certainly these may have negated some organ weight differences between SIDS and normal babies but head and brain size (and possibly thymus) are supported by recent data. It is well known that the thymus is relatively large in normal babies. Confusion continues with regard to whether or not this organ is bigger in SIDS than comparison deaths. Where sudden death (for example, through accidental injury is the basis for selection of the comparison group), thymus weights are significantly greater in SIDS babies [80]. Growth curves derived mathematically from such data indicate a prenatal origin of the larger thymus in SIDS [80]. Similarly, brain weight (and head circumference) are greater in SIDS cases than comparisons [81]. This feature is recognised by the National Institute of Child Health & Human Development (NICHD) [8].

The excessive brain weight might reflect abnormal cerebral development and could be detrimental to vital neural control. In a recent study, Kadhim *et al*. [82] revealed cytokine over-expression in the brains of SIDS victims. Whether increased brain weight is linked to cytokine up-regulation remains, however, moot and merits further exploration.

As alluded to in a previous section, cardiac and renal weights [53,54] may also be decreased in SIDS; this possibly reflects the effects of similar prenatal/developmental influences responsible for brain anomalies. Serial examination of the cerebral hemispheres of 20 sudden infant death syndrome victims revealed a high incidence of leukomalacia (40%), leptomeningeal glioneuronal heterotopias (70%) at the base of the cerebrum, and astrogliosis (65%) in the white matter and medulla reticular formation compared with 20 age-matched controls. These results suggest that an antepartum insult may become an important predisposing risk factor in some individuals for sudden infant death syndrome [83]. Kariks [84,85] noted histopathological changes in large proportions of SIDS babies' myocardium. He attributed the changes to shock which would be compatible with general pathological and epidemiological features of SIDS.

#### Liquid unclotted blood in the chambers of the heart

It is surprising that this common pathological finding has not been explored extensively. As far as the author is aware, the study by Goldwater *et al*. [86] is the only one to look at possible mechanisms underlying this suspected coagulopathy. The finding of significantly elevated fibrin degradation products (D-dimer) in blood from SIDS cases could reflect a perturbation of coagulation pathways brought on by NFkappaB-centered innate immune pathways and a shock-like demise.

#### An empty bladder and rectum

Infants meeting the currently accepted definition of SIDS are usually normally nourished and hydrated. The diapers are usually wet and contain stool. The bladder and rectum are typically empty [55,56]. Interpretation requires care. Plausible explanation would include bladder emptying reflecting an agonal event, or shock (toxic/septic/cardiogenic).

#### Laryngeal basement membrane thickness

The finding of vocal cord laryngeal basement membrane thickening by Shatz *et al*. [87] was refuted by Krous *et al*. [88] as not being a reliable marker for SIDS.

#### Inflammatory cells in airway, lung, and cardiac tissues

The work of Rambaud *et al. *[89] has revealed subtle evidence of acute inflammatory cells within airway, lung and cardiac tissue. Interpretation of these findings remains controversial. However, no consensus is possible regarding the degree of inflammation and evidence of infection.

Krous *et al. *[90] found numbers of lymphocytes, macrophages, and necrotic cardiomyocytes were not statistically different between a small cohort of SIDS and suffocation cases. Based on these findings the authors concluded that very mild myocardial lymphocyte and macrophage infiltration and scattered necrotic cardiomyocytes in SIDS are not pathologic, but may occur after the developing heart is exposed to environmental pathogens, including viruses. This is clearly somewhat questionable given that evidence of viral infection of tissue *per se *would be regarded as not normal and, therefore, pathological [91]. Dettmeyer *et al. *challenged Krous *et al. *by drawing attention to consensus difficulties of criteria for meeting a diagnosis of myocarditis. Dettmeyer *et al*. [92] subsequently showed that viruses when found in the myocardium are associated with inflammatory change and, therefore, these changes are pathologic. The changes were only found in myocardium from SIDS cases and were not found in unexpected unnatural sudden deaths.

Supportive evidence (such as would be obtained with gene expression of innate immune system genes and *in situ *hybridization of a wide range of microbial nucleic acids) should provide answers.

### Analysis of hypotheses

#### **Brainstem control of cardiorespiratory function**

SIDS deaths commonly occur during a sleep period. This has led to a dominant hypothesis that SIDS is due to abnormal brainstem control of cardiac and/or respiratory function [46-48]. A brain stem abnormality related to neuroregulation of cardiorespiratory or other autonomic functions has been proposed [48]. The hypothesis is based on autopsy studies indicating possible pre-existing, chronic low-grade hypoxemia attributed to sleep-related hypoventilation. The autopsy evidence for chronic hypoxemia includes (1): persistence of adrenal brown fat, hepatic erythropoiesis, brain stem gliosis and other structural abnormalities [52], and (2): evidence of hypodevelopment of brainstem structures [50] and multiple neurotransmitter abnormalities in brain stem regions relevant to neural cardiorespiratory regulation [93,94]. These post- mortem studies of neurotransmitters and receptors have identified reduced muscarinic cholinergic binding via 3H-kainate receptors, in the arcuate nucleus, a region of the human brainstem analogous to the ventral medullary surface involved in chemosensitivity to CO_2 _in laboratory animals [95,96]. SIDS victims also have been observed to have decreased binding of serotonin in the nucleus raphe obscurus, a brain structure linked to the arcuate nucleus, and in four other brain regions [94]. These brain regions have their origin in a part of the rhombic lip, which, during embryogenesis gives rise to structures in the brain stem considered important in regulating arousal responsiveness from sleep, as well as breathing, heart rate, and body temperature. Deficient kainate binding in the arcuate nucleus and deficient serotonergic receptor binding in the brain stem may reflect genetic changes resulting in abnormal prenatal development of the brain stem. It is possible, that these apparent brain stem abnormalities are secondary to "upstream" deficit(s) (for example, genetic mutation(s) or copy number variation, yet to be defined). Alternatively, neurotransmitter abnormalities may represent interaction(s) between environmental risk factors and susceptibility genes associated with autonomic dysregulation. Such an example could be smoke exposure and/or infection in pregnancy. Alternatively, these environmental influences could represent epigenetic changes through DNA methylation with consequent impact on gene expression.

Because apnea is commonly observed in neonates born at term or prematurely, a link with SIDS gained popularity. Apnoeic spells are typically accompanied by bradycardia and desaturation of oxyhaemoglobin. Such apnoeic spells are thought to reflect immaturity of cardiorespiratory control because these episodes predictably resolve as the infant matures. Nevertheless, several studies have shown that infants who have apnea in the newborn period are not at higher risk for sudden infant death syndrome (SIDS) [97]. Reconciling this would logically mean that brainstem changes found in SIDS babies are not associated with apnea *per se *and would enforce a conclusion that the anatomical/neurotransmitter changes observed may result in death through a different mechanism. It is, therefore, uncertain as to why research focusing on apnea continues to receive funding support. Several authors claim that hypoxia and/or hypercarbia (secondary to apnea) triggers abnormal physiological responses arising as a result of pathological brainstem abnormalities [98]. Studies of the brain tissue of SIDS infants and piglets have shown a number of similarities [99]. Using a piglet model, it was shown that post-natal period exposure to noxious stimuli that create known risk for SIDS can produce similar brain abnormalities in piglets to those seen in SIDS infants. Do these results support the hypothesis that an infants' vulnerability to SIDS need not be present before birth to still predispose the child to sudden death? The argument seems to be circular in that apnea *per se *is not correlated with SIDS yet the perception of risk is confused with histopathological brain changes observed in a proportion of SIDS cases [99].

The review by Poets [36] neatly sums up the evidence against an association between apnea and SIDS. This is based on objective data on the pathophysiological mechanisms that immediately precede SID obtained from physiological memory monitor recordings. Some 14 recordings have been published [35,58-60,100-102] Poets [36] concluded that none of the recordings showed evidence for the occurrence of prolonged central apnea as the primary cause of death. Instead, the primary event in every case was a progressive decrease in heart rate, which developed over minutes or hours. The exact cause(s) of the bradycardias could not be determined from the recordings, but there is evidence from similar recordings that have been obtained during apparent life-threatening events and included data on oxygenation that this progressive decrease in heart rate was probably due to hypoxic cardiac depression [10,103-105].

Moreover, no studies have showed that SIDS and idiopathic acute life-threatening events (ALTEs) are the result of the same mechanism or that an ALTE would result in death [106]. Fewer than 10% of SIDS victims have a history of a prior ALTE. In the United States, the multicenter Collaborative Home Infant Monitoring Evaluation (CHIME) study [107] was designed to assess several of the unanswered questions regarding home monitoring. The study, supported by the NICHD, had specific aims, which included the assessment and comparison of the incidence of clinically important events identified by documented monitoring in infants considered to be at risk for SIDS (including apnea of infancy patients, siblings of SIDS, preterm infants <1,750 g at birth) and normal infants. It also sought antecedent predictors of clinically significant events and determined the outcome of such events. The findings of this study of 1,079 infants and 718,358 hours of monitoring demonstrated that both conventional apnea and bradycardia and extreme apnea and bradycardia are relatively common events, even among healthy term infants. The only group that had an increased risk of such events compared with healthy term infants was preterm infants, and only up to 43 weeks' post-conceptional age. It is of note that the peak incidence of SIDS is more than 43 weeks' post-conceptional age for preterm infants of any gestational age. Therefore, the evidence suggests that prolonged apnea and bradycardia are not immediate precursors of SIDS. Thus, the CHIME study could not determine if infants who had episodes of extreme apnea or bradycardia were at higher risk for SIDS, nor could it determine whether or not home monitoring could provide warning in time for intervention. The study could not provide evidence that, or if, intervention would prevent unexpected death. Other epidemiologic studies have failed to document any impact of home monitoring on the incidence of SIDS. Given the lack of evidence that home monitoring has any impact on SIDS, *prevention of SIDS *should not be an indication for home monitoring. Home monitoring may be used to document apnea, bradycardia, or hypoxemia (depending on the monitor used), but there is no evidence that any of these events is associated with an increased risk of SIDS; the overwhelming evidence suggests that they are not. There is no evidence that monitoring will decrease the risk of SIDS in preterm infants who do or do not have apnea, siblings of SIDS victims, or infants who have had a prior ALTE. Evidence that siblings of SIDS victims or of infants who have had an ALTE are at any increased risk for SIDS is inconclusive and inadequate. In other words, there is no good evidence that either of these groups is at increased risk of SIDS.

### Respiratory obstruction

Upper respiratory obstruction has long been promulgated by Tonkin's [50] and Krous' [49] groups based on purported anatomical changes said to occur when a baby is placed prone. While some supportive evidence exists [51] this approach is flawed because such a proposed mechanism is undermined by the lack of evidence that asphyxiation is a plausible mode of death in SIDS [36].

Much has been written about the variety of mechanisms that come under the banner of asphyxia. These include smothering/overlaying and co-sleeping, obstruction of breathing by soft bedding/coverings, and central apnea. These mechanisms remain popular as a result of the important risk factor - a prone sleep position. Generally, compared with supine, the prone position raises arousal and wakening thresholds, promotes sleep and reduces autonomic activity through decreased parasympathetic activity, decreased sympathetic activity or an imbalance between the two systems. Resting ventilation and ventilatory drive is improved in preterm infants, but in older infants (>1 month), there is no improvement in ventilation, and in three-month-old infants. The prone position is associated with poorer ventilatory drive (in active sleep only). The majority of findings suggest a reduction in physiological control related to respiratory, cardiovascular and autonomic control mechanisms, including arousal during sleep in the prone position. The majority of these findings are based on studies of healthy infants [108]. Given the lack of evidence that breathing is the primary failing point in the pathogenesis of SIDS, we are, nevertheless, more inclined to agree on the effect of proneness on brainstem-cardiac control. We can then look at the risk factors that might trigger a fatal event in a baby with inherited or acquired susceptibility; standing out among these is infection. Also, supportive evidence for plausible links between prone sleep position and infection have been proposed based on bacterial colonization and toxin induction with raised nasopharyngeal temperatures prone [17], and bacterial contamination of the sleeping surface promoting colonization of the infant's nasopharynx and gut [16].

### Critical diaphragm failure

This hypothesis postulates that the cause of death in SIDS is respiratory failure caused by failure of the diaphragm because of a non-lethal infection occurring on a background of underdevelopment of respiratory muscles, prone position, and REM sleep [109]. Infection can reduce mitochondrial function of the diaphragm. Free-radical generation plays a central role in infection-associated myopathy. Melatonin (N-acetyl-5 methoxytryptamine) is produced by conversion from serotonin in the pineal gland. Melatonin is a highly effective free-radical scavenger and protects against oxidative stress. Infants show transient melatonin deficiency in the first few months of life. An association between pineal dysfunction and impaired melatonin metabolism with SIDS has been demonstrated [110]. Diaphragm fatigue and inactivation of intercostal muscles is observed during REM sleep and a prone position increases diaphragmatic work. Adding infection to the mix hypothetically could lead to SIDS.

### Triple risk hypothesis

The triple risk hypothesis, while containing plausible elements in the causation chain (vulnerability, age-specific risks, and precipitating factors), falters on arguments centered on the origin of brainstem pathology, and its failure to adequately deal with other key aspects of pathology (for example, intrathoracic petechiae, and so on). Key protagonists of the hypothesis, Guntheroth and Spiers (2002) [14] appear to discard evidence that the brainstem abnormalities have a prenatal origin and remain attendant to hypoxia as the underlying cause. The supposed evidence of hypoxia is based on abnormal retention of periadrenal brown fat, increased hepatic erythropoiesis, brain stem gliosis, abnormally thickened smooth muscle walls of small pulmonary arteries, hypertrophy of the free wall of the right ventricle, increased chromaffin tissue in the adrenal medulla, and either hypoplasia or hyperplasia of the glomic tissue in the carotid body [111,112]. Most of the changes appear to be age-related (immaturity-associated) and because SIDS is associated with low gestational age the changes observed could well be a manifestation of this rather than a finding specific to SIDS.

### SIDS epidemiology

#### Risk factors

The epidemiological risk factors for SIDS can be divided into 1) genetic predisposition, 2) prenatal influences and 3) post-natal risks. For the purposes of this review, these have been summarized below:

#### Risk factors for SIDS

1. Demographic factors and genetic predisposition

• Ethnicity [113]

• Low socio-economic status [114]

• Gender (and possible X-linked genetic mutations/copy number variations, and so on) [115]

• Genetic control (recently reviewed by Courts and Madea [115] and Opdah [116]

■ Genetic control of inflammatory response [113,116]

■ Genetic control of NOS1 [117]

■ Genetic control of brainstem function [118]

■ Genetic control of metabolic pathways, for example, flavin-monooxygenase 3 (FMO3) an enzyme metabolising nicotine [119]

■ Genetic control of cardiac function [117,118,120-122]

#### Prenatal risks

• Maternal smoking/nicotine use [119,123]

• Inadequate prenatal care [124]

• Inadequate prenatal nutrition [125]

• Use of heroin, cocaine and other drugs [126]

• Subsequent births less than one year apart [127]

• Alcohol use [126]

• Infant being overweight [128]

• Mother being overweight [128]

• Teen pregnancy (if the baby has a teen mother, it has a greater risk) [129]

• Maternal anemia:[130]

#### Post-natal risks

• Seasonality [131]

• Viral respiratory or gastrointestinal symptoms in the days before death [132]

• Low birth weight (in the US from 1995 to 1998 the rate for 1,000 to 1,499 g was 2.89/1,000 and for 3,500 to 3,999 g it was 0.51/1,000) [133]

• Exposure to tobacco smoke [119]

• Prone sleep position (lying on the stomach, see sleep positioning below) [134]

• Not breastfeeding [135]

• Elevated or reduced room temperature [136]

• Excess bedding, clothing, soft sleep surface and stuffed animals [137]

• Co-sleeping with parents or other siblings may increase risk for SIDS, but the mechanism remains unclear [138]

• Sofa-sleeping [139]

• Infant's age (incidence rises from zero at birth, is highest from two to four months, and declines towards zero at one year) [140]

• Prematurity (increases risk of SIDS death by about four times) [133]

• Probable anemia [130,141] (haemoglobin cannot be measured *post mortem*)

## Conclusion and final analysis

Table 2 summarizes the hypotheses and their relationship to how they account for pathological findings, epidemiological risk factors and whether or not the hypotheses link pathology and epidemiology. It is clear that most hypotheses fall well short of this measure. On the basis of this apparent shortfall it is, therefore, incumbent upon all future research to take formal measures to simultaneously address both pathological and epidemiological findings. This will go a long way to redress the oft misguided research that has ignored salient aspects of these and focused on usually a single mechanism (such as respiratory obstruction) and which has been undertaken at considerable expense to the public purse.

**Table 2:** Final analysis

Hypothesis	1. Does the hypothesis take into account the key pathological findings?	2. Is the hypothesis congruent with the key epidemiological risk factors?	3. Does the hypothesis link 1 and 2?
Brainstem control of respiratory function	No	No	No
Brainstem control of cardiac function	Partly	Yes	Partly
Respiratory obstruction/unintentional asphyxia	No	Partly	No
Common bacterial toxins	Yes	Yes	Yes
Shock including anaphylaxis	Yes	Yes	Partly
Thermal stress	Partly	Yes	No
Diaphragm failure	Partly	Yes	Partly
CO2 rebreathing	No	No	No
toxic gases emitted from fungally colonized mattresses	No	No	No
Abuse	No	No	No

## Future directions

The investigation into the pathogenesis of SIDS deserves a return to fundamental problem-solving so that each hypothesis is required to account for all aspects of the syndrome, especially the pathological findings and epidemiological risk factors. Research should not be conducted without this holistic approach. Tens of millions of dollars of research funding has been channelled into dead-end investigations because simple balanced enquiry has been sacrificed. Indeed, I am aware that some researchers rely on funding on the basis of defunct hypotheses because they know that their granting bodies whose organ system-based *raison d'être *would not continue the dollar supply if the hypothetical point of view was made more evidence-based.

The future holds great promise in discovering the key genetic predisposing elements and the prenatal contributors underlying SIDS, but novel approaches should be readily taken up to facilitate discovery. The integrated modelling of Salomonis *et al*. [142] (2010) goes some way toward this goal. The model provides an integrated view of SIDS at the level of implicated tissues, signaling networks and genetics and acknowledges the key aspects and clue areas (tissue pathology, genetic predisposition in terms of neuronal signaling, cardiac function and inflammatory responses to viral and bacterial antigens). The purpose of this model is to serve as an overview of research in this field and recommend new avenues for more focused or genome-wide analyses. While no clear diagnostic markers currently exist, several polymorphisms have been identified which are significantly over-represented in distinct SIDS ethnic populations. The large majority of these polymorphisms exist in genes associated with neuronal signaling [116], cardiac function [143] and responses to infection/inflammatory response [144]. The genetics of SIDS is well reviewed by Courts and Madea [115] and Opdal and Rognum [116]. The role of testosterone also deserves close examination because of the hormone's profound effect on immune function [145] and most notably the hormone's peak observed in male infants between the age of two and four months which coincides with the SIDS age peak [146].

Both genetics and proteomics (introducing new mass spectrometry technologies such as SELDI-TOF MS) offer enormous opportunities for solving the enigma of SIDS pathogenesis. Because males are at higher risk of both SIDS and most infectious diseases than females [147], the X-chromosome would be a logical site to explore associations between SIDS and possible X-linked SNPs involved in innate immune responses [148,149]. Also, the linchpin of the innate immune response, the transcription factor NF-kappaB, is activated by a wide variety of stress signals including proinflammatory mediators, and regulates expression of genes with immune and inflammatory and anti-apoptotic functions. One such regulatory protein is X-chromosome-linked inhibitor of apoptosis (XIAP) that, besides its anti-apoptotic properties, has been shown to enhance NF-kappaB activity. XIAP interacts with and ubiquinates MEKK2, a kinase associated with biphasic NF-kappaB activation [149]. Logically, SNPs in XIAP would be obvious targets. Toll-like receptor pathways are important in early innate immune responses. The TLR8 gene is located on the X-chromosome and is, therefore, another suitable site to explore. There are several stable polymorphisms (SNPs) in the TLR8 gene, one associated with an increased risk of infection in males is rs3764880 (met > val), another target SNP to investigate [150]. Because many clues indicate the heart could be the final weakness in the SIDS story, the work of Sartiani *et al*. [151] could confirm a link between smoking in pregnancy, fetal exposure to carbon monoxide and abnormal cardiac development.

The old problems will continue to bother us; accessing tissues and finding suitable properly matched controls will remain a significant problem. When the answers eventually fall into place, and if the inherited ones include defects in pathogen recognition, we may have an opportunity to enhance prevention strategies with old strategies, such as active/passive immunization of infants and/or pregnant mothers. Alternatively, concerted efforts to eradicate the scourge of smoking (especially in pregnancy) by considering that it be legislated as a form of child abuse would benefit the lives of so many babies, their mothers and the community.

## Abbreviations

ALTE: acute life-threatening event; CHIME: Collaborative Home Infant Monitoring Evaluation; CODE: cause of death established; CL: confidence limits; NICHD: National Institute of Child Health & Human Development; OR: odds ratio; REM: rapid eye movement; SELDI-TOF MS: surface enhanced laser desorption and ionization time-of-flight mass spectrometry; SIDS: sudden infant death syndrome; SUDI: sudden unexplained death of infancy; SNPs: single nucleotide polymorphisms; TLR: Toll-like receptor.

## Competing interests

The author declares that he has no competing interests.

## Author's information

Prof. Goldwater has been involved in SIDS research for over 30 years. He is a senior consultant clinical microbiologist and infectious diseases physician, and with his collaborators has used his professional knowledge to further develop an understanding of the important role of infection in sudden unexpected death in infancy.

## Pre-publication history

The pre-publication history for this paper can be accessed here:

http://www.biomedcentral.com/1741-7015/9/64/prepub
